# Phytoremediation of pollutants from wastewater: A concise review

**DOI:** 10.1515/biol-2022-0056

**Published:** 2022-05-13

**Authors:** Atta Ullah Khan, Allah Nawaz Khan, Abdul Waris, Muhammad Ilyas, Doaa Zamel

**Affiliations:** CAS Key Laboratory of Standardization and Measurement for Nanotechnology, CAS Center for Excellence in Nanoscience, National Center for Nanoscience and Technology, No. 11 Zhongguancun Beiyitiao, Beijing 100190, China; Department of Biotechnology, University of Malakand, Pakistan; University of Chinese Academy of Sciences, Beijing 100049, PR China; Department of Botany, University of Faisalabad, Pakistan; State Key Laboratory of Vegetation and Environmental Change, Institute of Botany, Chinese Academy of Sciences, Xiangshan, Beijing, China; Department of Biomedical Sciences, City University of Hong Kong, Kowloon Tong, Hong Kong SAR; Department of Biochemistry, Faculty of Science, Helwan University, Helwan, Egypt; Department of Environmental Engineering, Institute of Urban Environment, CAS, China

**Keywords:** plants, pollutant removal, phytoremediation, wastewater treatment

## Abstract

As there is a global water crisis facing the whole world, it is important to find alternative solutions to treat wastewater for reuse. Hence, plants have an effective role in removing pollutants from wastewater, which has been emphasized in this review article. Biological treatment of wastewater can be considered an eco-friendly and cost-effective process that depends on in the future. Living organisms, including plants, can remediate pollutants in wastewater, especially in agricultural fields, such as dyes, heavy metals, hydrocarbons, pharmaceuticals, and pesticides. This review discusses the different activities of plants in pollutant elimination from wastewater and sheds light on the utilization of plants in this scope. This review focuses on the remediation of the most common contaminants present in wastewater, which are difficult to the removal with microorganisms, such as bacteria, fungi, and algae. Moreover, it covers the major role of plants in wastewater treatment and the potential of phytoremediation as a possible solution for the global water crisis.

## Graphical abstract

**Figure:**
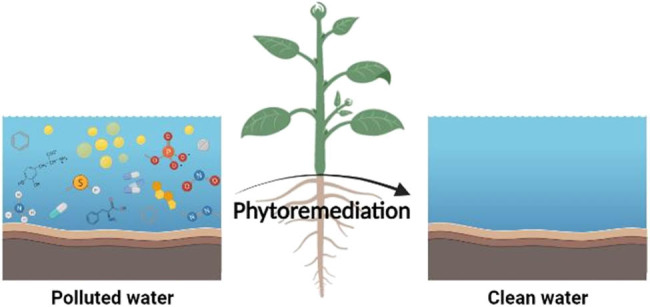

## 1 Introduction

Wastewater is the most threatening to the localized environment where the untreated water discharges fluently, which causes many problems in controlling the challenges of supplying clean water to rural and urban regions [1]. Water pollution problems are mainly caused by the effluents of wastewater, which leads to eutrophication. However, these may stimulate algal growth, higher purification costs, health risks to livestock and humans, and excessive oxygen loss, which may cause various changes in the population of aquatic systems [2,3,4]. Numerous hazardous chemical and non-chemical compounds have been introduced by mankind to the environment in the era of industrialism. Pollutants include dyes, heavy metals, organic compounds, and inorganic compounds of hazardous nature, which can pose severe risks to human health. Various techniques and methods may be used to prevent, remove, and correct the negative impacts of pollutants released into the environment. Reducing the contaminant level in soil plants can be used as a cost-effective method that reduces the risk to the ecosystem and human health damaged by contaminated sites [5,6,7]. The presence of harmful chemicals in water has a detrimental impact on the water environment by obstructing light penetration, which stops aquatic plants from photosynthesis [8]. The impact of toxic metal ions may be reduced by various methods, such as membrane filtration, reverse osmosis, chemical precipitation, oxidation, adsorption, and flotation. However, adsorption is very accurate and common due to the uptake of metal in low concentrations, which has feasible economic properties [9,10]. The plants primarily take up the contaminants by the root system, leading to the prevention of toxicity. Besides, root systems come up with a large surface area that accumulates and absorbs the essential nutrients and water for growth along with non-significant contaminants, which help to remediate the contaminants from wastewater and make it clean [11,12]. During experimental studies, it has been proven that the *Salvinia molesta* plant and others have a greater ability to remove the toxic dyes and contaminates; now, it is a promising approach that is being used in different industries [13,14]. Plant-based green technology and adsorption treatments are common treatments for the removal of contaminates. A lot of progress in this field has been documented in the past few years [15,16]. Different adsorption capacities, operating circumstances, and application forms through experiments revealed that biomass adsorbents are extremely influential and recyclable. Plant components such as the leaf, peel, and other parts efficiently remove contaminants [17,18]. The current review is here to discuss the role of plants in the removal of dyes, heavy metals, inorganic elements, pesticides, hydrocarbons, and pharmaceutical containments from the wastewater. Furthermore, it emphasizes the crucial role played by plants in this area, and recent publications have addressed it. This review addresses a novel discussion on the phytoremediation of dyes, heavy metals, hydrocarbons, pharmaceuticals, inorganic elements, and pesticides, which are the most abundant pollutants in wastewater.

## 2 Various phytoremediation methods

**Figure 1:**
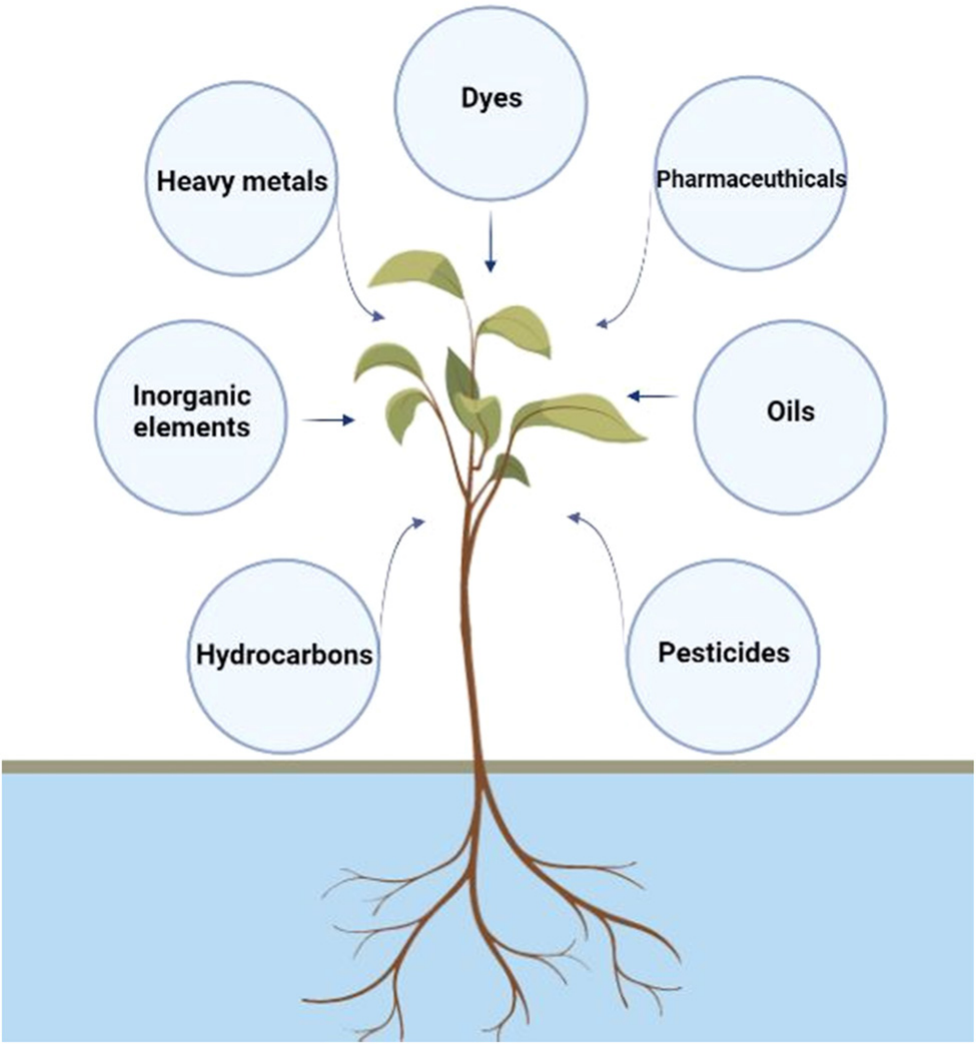
Different roles of plants in the removal of pollutants.

Phytoremediation technology is an emerging green approach used to detect, degrade, and remove various types of pollutants from the environment. Different types of contaminants that cause harmful effects on human health and other biological systems are removed using plant species. These plant species uptake these pollutants from the environment and detoxify their toxic effect. Because of its eco-friendly nature, this approach has an advantage over the traditional techniques, which cause harmful effects on the biological system and environment [6]. Several mechanisms are involved in the remediation of pollutants from water, especially metal contaminants, to convert these into nontoxic compounds, leading to the removal of waste from water. These mechanisms include phytostabilization, rhizodegradation, phytofiltration (also called rhizofiltration), phytoextraction, photodegradation, phytovolatilization, and phytoaccumulation, as shown in Table 1 [6,19]. In phytostabilization, the mechanisms of accumulation or adsorption are used. In this approach, the contaminants in the groundwater or soil are adsorbed onto the root or accumulate in the rhizosphere to prevent the movement of pollutants from one place to another in the environment [20]. While in the case of rhizodegradation, the pollutants, especially the heavy metals and organic wastes, are degraded and breakdown in the rhizosphere and converted into none or less toxic compounds. This process is also enhanced by using various types of microorganisms [21]. The rhizofiltration approach also used the same mechanism as the phytoremediation. However, the pollutants are absorbed by the plants’ roots in this case. Phytodegradation leads to the degradation of wastes or pollutants through metabolic ways. In this process, the plant uptakes the metals or waste from the environment or wastewater and degrades them into nontoxic compounds with the help of different enzymes. This process is also known as phytotransformation [22]. In the phytovolatilization approach, plants absorb different types of wastewater and convert them into nontoxic compounds. Later, these nontoxic compounds are released into the atmosphere using leaves by the process of transpiration. Similarly, when the wastes are stored in various parts of plants, such as roots, shoots, and leaves, this process is termed phytoaccumulation [6,19]. Different types of plant species have been used for the removal of different types of heavy metals, organic wastes, and other types of contaminants (Figure 1).

**Table 1:** Various techniques for phytoremediation

Technique	Application	Containment	Mechanism	Description	Accumulation part	Ref
Phytodesalination	Soil	Organics salt	Salt reduction by conversion	Removal of salts from soils by halophytes	In plant tissues	[23,24,25]
Rhizodegradation	Soil	Inorganics organics	accumulation in rhizosphere	Degradation of organic through rhizospheric microorganisms	Rhizosphere	[26,27]
Phytofiltration rhizofiltration	Water	Organics inorganics heavy metals	Adsorption absorption	Pollutants uptake from contaminated waters by aquatic plants	Aerial parts or roots	[28,29]
Phytodegradation phytotransformation	Soil and water	Organics	Degradation in plant rhizosphere	Organic degradation by plant enzymes	In plant tissues	[28,30]
Phytoextraction phytoaccumulation	Rare in water and soil	Heavy metals and inorganics	Hyperaccumulation	Accumulation of pollutants in by root and translocate them to upper parts	Shoots	[26,31]
Phytostabilization	Water and soil	Heavy metals and inorganics	Precipitation sorption complexation	Mobility limitation pollutant accessibility in soil by plant roots	Reduction in rhizosphere	[26,32]
Phytovolatilization	Water and soil	Various heavy metals and organics	Volatilization by leaves	Conversion of pollutants to volatile form	Release to the atmosphere	[33,34]

## 3 Removal of pollutants from wastewater by plant species

### 3.1 Removal of dyes

The presence of harmful chemicals in water has a detrimental impact on the water environment by obstructing light penetration, which hinders aquatic plants from photosynthesis [8,35]. During experimental studies, it has been proven that the *S. molesta* plant has a greater ability to remove the toxic dyes, and now, it is a promising approach that is being used in different industries [13]. In recent years, single or combined processes, such as ultrasonic, electrochemical, photochemical, and membrane separation, got much attention therefore known as state of the art technologies applied to remove toxic dyes found in wastewater [36]. There are various chemical, physical, and biological approaches to remove color dyes [37]. The oxidation process includes H_2_O_2_, cavitation, ozone, and Fenton, while physical and biological methods include adsorption, filtration, plants, fungi, bacteria, and algae [38]. Plant-based green technology and adsorption treatments are common treatments for removing dyes. Much progress in this field has been documented in the past few years [15]. Phytoremediation is primarily a plant-based approach in which dyes from textile sources are removed through plants, and it is a relatively new field of study [39]. Different adsorption capacities, operating circumstances, and application forms through experiments revealed that biomass adsorbents are extremely influential and recyclable. Plant components, such as the leaf, peel, and other parts, are quite efficient in removing dyes [17]. Textile dyes treated with plants are a new concept. Chemicals, such as Acid Orange 7 and toxic sulfonated anthraquinones, are effectively removed with macrophytes. Many ferns and other plant species have succeeded in the removal of dye including *Aster amellus*, *Glandularia pulchella*, and *Zinnia angustifolia* [40]. On aminated and non-aminated seed hulls of common sunflower, the sorption of popular textile dyes (Reactive Black 5, Reactive Yellow 84, Acid Yellow 23, and Acid Red 18) was tested (*Helianthus annuus* L.). The fourier-transform infrared spectroscopy (FTIR) analysis was performed on the sorbent. When compared with non-modified hulls, the aminated sunflower seed hulls had a greater sorption capacity against reactive dyes [41]. Using parameters, such as pH, agitation rate, contact time, and sorbent dosage using a batch process, the role of lignocellulosic residues and pineapple plant stem shows greater affinity for cationic dyes. The capacity of polyphenylene sulfide (PPS) to regenerate using acid suggested that it might be used as a bio-sorbent for BB3 elimination [42]. There are almost four types of adsorbents that are biomass derived. Rice ash, tea waste, and pineapple leaf are widely used adsorbents due to their low cast and extraordinary efficiency in dye removal [43]. The residue of agroindustry like leaves of *Cucurbita moschata* along with stalks of *Beta vulgaris* showed great potential for removing tetrazine dye and red dye of Bordeaux [44]. The efficiency of *Eichhornia crassipes* was excellent for removing color dyes when treated with a 50 mg L^−1^ concentration of methyl orange dye for 20 consecutive days at a temperature of 30°C [45]. When exposed to the dye, the oxidative and anti-oxidative properties of *Bacopa monnieri* plants show greater efficiency in roots and other vegetative parts. The presence of enzymes in these parts of plants has a high recommendation for industrial usage [46].

### 3.2 Removal of heavy metals

Heavy metals have serious threats to the environment and ultimately hazardous effects on human health. Different techniques have been developed to remove heavy metals but are expensive compared to the plant-based removal of toxic metals in industries [47]. Heavy metal pollution is becoming more of a problem in developing countries. Many studies have been done on less expensive and eco-friendly (plant-derived) adsorbents in removing heavy metals [48]. Heavy metal accumulation in air, soil, and water has become a global issue due to anthropogenic activities, such as mining, urbanization, and industrialization. Due to their multiple roles in the environment, ornamental plant species are highly suggested [49]. Industries are polluting soil and water with their effluent discharges without cleaning. These effluents contain heavy metals, which are now being cleaned up by plant-based techniques (phytoremediation) [50]. Wastewater heavy metal contamination is a major danger for aquatic life. Phytoremediation is a cost-effective, environmentally friendly, developing technique with long-term use. For the removal of heavy metal contaminants, aquatic plants have high efficiency. Duckweed (*Lemna minor*) and a few other plants have great metal accumulator efficiency [51]. For wastewater treatment, heavy metal adsorption through low-cost and environment-friendly adsorbents like plants is preferred. Residues of natural lignin isolated through black liquor are best for use as bioadsorbents [52]. Biosorption is an effective methodology to remove heavy metals and other toxic substances present in the environment and has been widely investigated in the past through multi-research [53]. Plant-based waste for metal removal in industrial wastewater has sparked a lot of interest because of its cost-effectiveness and a significantly greater removal rate, which may be ascribed to various functional groups. Coconut wastes and bark of black oak can remove metals, such as lead, cadmium, and mercury [54]. Trunk fiber waste of different date palm varieties for the removal of heavy metals shows excellent results, but project on massive scales is still understudies due to its cost-effectiveness and availability [55]. Plants are used in various sectors to remove heavy metals from contaminated soil, recover metal-contaminated habitats, and prevent ongoing environmentally harmful effects on living organisms [56]. Using green plants over different conventional techniques is more effective in removing heavy metals from soil and wastewater [28]. Heavy metals can be removed from wastewater efficiently by using various methods, including ion exchange, electrolysis, adsorption, membrane filtration, and coagulation [57]. Chemicals extracted from plants and many microorganisms act as removal agents for heavy metals [58]. Metal-removing capacity of the hyacinth (*E. crassipes*) plant using atomic absorption spectroscopy shows great potential for mercury, cadmium, and arsenic [59]. Physical aspects and chemical and leaching properties of dry *Miscanthus* (silver grass) plant, analyzed through surface brunaur-emmett teller method (SBET), differential scanning calorimetry (DSC), X-ray diffraction (XRD), Thermo gravimetric analysis (TGA), inductively coupled plasma (ICP), and elemental analysis, show the highest degree of potential to clean heavy metals in wastewater [60]. The common problems caused by heavy metals’ presence in wastewater pressured the researchers to search for many ways of heavy metals’ remediation from wastewater. Here, phytoremediation proved their effective role to help in the elimination of heavy metals in wastewater.

### 3.3 Removal of inorganic elements

Large quantity of waste that is being produced all over the world, there is a huge demand for low-cost and ecofriendly adsorbents [61]. Many harmful chemicals are released into the environment due to large-scale industrial and domestic wastes. Many primary and secondary stage wastewater treatment processes are being exercised to remove toxic chemicals in wastewater [62]. Coagulation is the conventional method that is used for wastewater treatment purposes [63]. Conventional treatments are expensive and inadequate. Green technology provides the best methods at a low cost within a healthier environment. *Opuntia ficus-indica* has maximum sorption capabilities for pollution [64]. Constructed wetlands are best due to low-cost, environment-friendly wastewater treatments that are exercised to remove toxic chemicals on a larger scale [65]. Food security problems that are being raised in the twenty first century are majorly due to phosphorus. Well-established wastewater treatment systems of plants are major areas to recover phosphorus at a low cost [66]. Phosphate is a very important part of fertilizers. Agriculture sectors are producing excessive phosphate as effluent. The roots of tomato plants are much more efficient in removing excess inorganic wastes [67]. Nowadays, much progress has been made in removing wastewater by phytoremediation [68]. Plants and microorganisms are highly involved in the nitrogen removal [69]. Biochar in its natural form holds the adsorption properties of phosphorus [70]. Contamination level has been increased in water bodies due to domestic and industrial effluents. Water hyacinth (*E. crassipes*) and other plants have a great capacity for removing organic and inorganic pollutants [71]. Plants like *Phragmites australis* with strains of certain bacteria have been investigated to remove inorganic elements from oilfields and wastewater [72]. Several aquatic plants have greater absorbent powder to eliminate toxic inorganic elements; *Pistia stratiotes* along with *S. molesta* is being widely introduced for large-scale industries due to their biomass efficiency [12]. Fungi and bacteria, along with plants, play an important role in phytoremediation. Fungi have a critical role in the transformation of organic and inorganic elements [73]. *Lactuca sativa* L., *Centaurea cyanus* L., *H. annuus*, and *Silybum marianum* Gareth have the extraordinary potential of removing very hazardous radionuclides that are fatal for life forms [74]. Major improvements have minimized inorganic wastes from wastewater. Multiple wastewater treatment technologies are at their peak of efficiency; still, there is more demand for low-cost, eco-friendly processes to remove toxic chemicals, such as phosphorus and nitrogen, present in wastewater [75].

### 3.4 Removal of pesticides

The use of pesticide products for agricultural activities is the major reason behind water contamination [76]. Due to its high use in agricultural fields, it has a high adverse effect on the ecosystem and aquatic organisms [77]. It is important to develop innovative technologies to clean the contaminated water and minimize this pollution. Present techniques for water remediation are based on physical and electrochemical treatments [78]. These techniques are very effective, but sometimes they produce hazardous by-products and are expensive. In the past ten decades, phytoremediation has gained popularity because of its environmentally friendly nature, cost-effectiveness, and *in situ* use to treat different types of pollution [79]. Removal of different types of pollution by phytoremediation involves a four-step mechanism. Direct uptake of contaminants accumulates pollutants in plant tissue for metabolization. It uses the transpiration method to remove different volatile organic hydrocarbons through leaves, releasing exudates from a different plant to remove different pollutants. These exudates activate microbial activity associated with plant roots, such as *mycorrhizal* fungi and microbial *concortia*, which remove different pollutions [80]. Different aquatic plants are used to treat water pollution, such as *E. crassipes*, *L. minor*, and *Elodea canadensis*, thanks to their high absorption of pollutants and high photosynthetic activity, ease of harvest, and high growth rates [81]. *E. canadensis* are macrophyte aquatic plants with fast growth and free floating in water considered weeds and native to North America. This species has great capability of removing water pollution and is commonly used in phytoremediation technology for the treatment of pesticides. Olette et al. investigated using *E. canadensis* to eliminate three different types of pesticides, such as dimethomorph (fungicides), copper sulfate (fungicides), and flazasulfuron (herbicides). All have the same toxicity for aquatic plants and occurred in the descending order such as flazasulfuron > copper sulfate > dimethomorph. The remediation percentage for copper sulfates in *E. canadensis* was 16.5%, and the remediation percentages for dimethomorph were 5.5% within 2 days and 12% within 4 days [79]. *E. crassipes* are aquatic macrophytes also known as water hyacinths that rapidly grow in high depth (over 60 kg m^−2^) open ponds and water bodies and cause clogging of water bodies [82,83]. Xia and Ma [13] performed an experiment using *E. crassipes* to eliminate ethion and malathion from polluted water in an aqueous solution. It was identified that 56% of 10 ppm of malathion (about 250 mL) is degraded by *E. crassipes*. Recent research showed that *E. crassipes* has greater capability in the uptakes and degradation of pesticides, which is potential and less expansive method for the removal of pesticides from water [84]. *L. minor* is another aquatic macrophyte, also known as duckweeds. It has a great ability to grow within 1 week when growing at pH 6 and also can withstand cold weather (1.7–35°C) [85]. These plants can decontaminate organic pollutants such as pesticides and heavy by the rhizofiltration method, which is cost-effective [81]. Dosnon-Olette et al. conducted research and used two species of duckweed plants to remove fungicides like dimethomorph from agricultural wastewater. The result showed that duckweed plants have a greater ability to eliminate dimethomorph [86].

### 3.5 Removal of pharmaceuticals

In recent years, in environmental chemistry, the occurrence of pharmaceutical compounds in the aquatic environment has been recognized as one of the emerging issues. Worldwide, Pharmaceuticals and their metabolites are detected in wastewater, groundwater, and even drinking water [87] Dordio et al. developed microcosm constructed wetlands systems established using both matrix of light expanded clay aggregates (LECA) and planted *with Typha* spp. The ability of *Typha* spp. to remove pharmaceuticals from wastewater, such as ibuprofen (IBU), carbamazepine, and clofibric acid, is investigated. Also, the seasonal variability of this system was determined. The result showed the removal efficiency of 96, 97, and 75% for IBU, carbamazepine, and clofibric acid, respectively, released in summer conditions after a retention time of 7 days. For clofibic acid, the removal efficacy is observed to be 26% in winter. Removal kinetics was also characterized by a fast-initial step (>50% removal within 6 h). Due to adsorption on LECA, the plant significantly contributed to system performance, but further tests using a larger-scale system are required for possible application of this system for wastewater treatment for dealing with pharmaceutical contaminated water [87]. Zhang et al. developed a system by using four wetland plant species which are commonly used in constructed wetland systems *Typha, Phragmites, Iris,* and *Juncus* to remove IBU and iohexol (IOH) from spiked cultural solution and to determine the mechanisms for the removal of this system. IBU are completely removed in 24-day experiment by all plant species, but IOH removal showed variation between 13 and 80%. *Typha* and *Phragmites* showed an efficient result in removing IOH and IBU. The first-order rate is observed at 0.38 and 0.06 day^−1^. These pharmaceuticals are completely taken by the roots of these plants and translocated to their aerial tissues [88].

### 3.6 Removal of hydrocarbons

Petroleum refineries and petrochemical industries released different hydrocarbons, such as polycyclic aromatic hydrocarbons (PAHs), which are major wastewater pollutants. These PAH compounds cause several health issues due to their benzene structure, mostly mutagenicity, carcinogenicity, and teratogenicity [89,90] Therefore, safe and effective remediation technologies are required to remove these pollutions from industrial wastewater. Phytoremediation is a new method to remove these hazardous pollutants, such as PAHs. Phytoremediation among the available process is a new method in which plants are used for the cleaning of water pollution because plants have high interaction with water bodies, media, and microorganisms; furthermore, plants have a positive role in the removal of contaminants from water [91]. Alsghayer et al. developed a procedure for horizontal subsurface flow-constructed wetlands for the removal of water waste, which consists of a high concentration of PAHs (phenanthrene, pyrene, and benzol[a]pyrene); for this purpose, the study used two plants namely *Vetiver* and *Phragmites*. The investigative parameters were designed (1) plants’ uptake of PAHs, (2) efficiencies of PAHs’ removal, (3) accumulated PAHs in the soil of crawling wave sonoelastography (CWs), (4) concentration factor of root/shoot, (5) translocation factor, and (6) correlations of PAHs to lipids present in plants. The results showed during the treatment period, that the highest concentration of phenanthrene in the shoot and the root systems of *Phragmites*, was 229.3 and 192 µg g^−1^; Pyrene was 69.1 and 59.2 µg g^−1^; and Benzo *[a]*pyrene 25.1 and 20.2 µg g^−1^, respectively and the *Vetiver* shoot and root system contains phenanthrene 87.5 and 64.1 µg g^−1^, Pyrene 63.2 and 42.1 µg g^−1^; and Benzo[a]pyrene 21.3 and 27.3 µg g^−1^, respectively [91].

## 4 Conclusion and future perspectives

To conclude, plants proved their effective roles in the remediation of pollutants in wastewater, which could be considered a crucial role depending on them in the near future. Scientists can assess the plants’ role in removing contaminants from wastewater, which cannot be achieved by small- and microorganisms. New avenues of phytoremediation have been addressed in this review on several types of contaminants that exist in large quantities in wastewater and have harmful effects on the environment and human health. Scientists need to think about integrating microorganisms with plants to enhance their efficiencies as microorganisms have an old history of pollutant biodegradation and remediation. Currently, there are no reports on the applications of plants for radioactive element removal; these ideas might be beneficial for researchers.
